# Vaccination in pregnant and postpartum women: Number 6 - December 2020

**DOI:** 10.1055/s-0040-1722522

**Published:** 2020-12-21

**Authors:** Giuliane Jesus Lajos, Susana Cristina Aidé Viviani Fialho, Renato de Ávila Kfouri, Renata Robial, Cecília Maria Roteli-Martins

**Affiliations:** 1 Universidade Estadual de Campinas, Campinas, SP, Brazil; 2 Universidade Federal Fluminense, Niterói, RJ, Brazil; 3 Faculdade de Ciências Médicas de Santos, Santos, SP, Brazil; 4 Fundação Faculdade Medicina, São Paulo, SP, Brazil; 5 Faculdade de Medicina do ABC, Santo André, SP, Brazil

## Key points

- Alert gynecologists and obstetricians about the importance of vaccinating pregnant and postpartum women;
- Address immunological changes in pregnancy and the puerperal period and susceptibility to infections;
- Highlight the maternal and fetal advantages of vaccination during pregnancy and lactation and offer tools that help obstetricians to convince their patients to adhere to vaccination;
- Describe the vaccines routinely recommended during pregnancy and puerperal period;
- Address vaccines given sporadically during pregnancy and puerperal period;
- Alert about contraindicated vaccines during pregnancy and inform risks of inadvertent use;
- Address future perspectives on the vaccination of pregnant women.

## Recommendations

- In pregnancy and the puerperal period, the practice of vaccination is essential, aiming at maternal and conceptus health. It should be part of the checklist of recommendations of gynecologists and obstetricians.
- Changes in cellular immunity lead to a greater susceptibility of pregnant women to infections such as influenza, with progression to severe forms of the disease. At the same time, pregnant women respond effectively to vaccination and produce antibodies appropriately.
- When pregnant women are vaccinated, there is a significant improvement in maternal and newborn health related to these infections. Note that by vaccinating the pregnant woman, the conceptus is protected because of the transplacental passage of antibodies. This is also true for breast milk. Perhaps this is the best argument for increasing adherence to immunization.
- Vaccines recommended during pregnancy are: influenza, dTpa (diphtheria, tetanus and pertussis) and hepatitis B (if the three doses have not been received previously).
- In some clinical or epidemiological conditions, inactivated vaccines, such as hepatitis A, pneumococcal and meningococcal viruses, may be recommended. These inactivated vaccines are indicated if there is a high risk of infection by these agents.
- The yellow fever vaccine, although of live attenuated viruses, may be given in pregnancy if the epidemiological risk justifies it. The use in women breastfeeding babies under six months is contraindicated.
- Triple viral, HPV, chickenpox and dengue vaccines are contraindicated during pregnancy. In case of inadvertent use, pregnancy must be maintained because the potential risks do not justify extreme measures.
- Vaccines for respiratory syncytial virus and group B streptococcus for specific use in pregnant women are in an advanced stage of development.

## Clinical context

While death is the most extreme consequence of vaccine-preventable infections, there are hundreds of thousands of secondary hospitalizations due to these diseases each year. 1

Immunological and physiological changes during pregnancy cause greater susceptibility to infectious conditions with increased morbidity and mortality, 2 as occurred in the influenza A-H1N1 pandemic in 2009. 3 When a pregnant woman acquires an infection, in addition to her health , there is a risk for compromising the health of the conceptus, for example, malformations, delayed intrauterine growth, premature birth, neonatal and late infectious manifestations, and even death.

In this sense, immunizing the pregnant woman is the primary action to prevent harm to the binomial. 4 Since more than 90% of pregnant women in Brazil attend prenatal consultations seeking care to ensure their health and of their conceptus, this is a unique moment to guarantee the vaccination of women and their family in a broad health promotion strategy. 5

In general, seroconversion by immunization during pregnancy is similar to that of women outside the pregnancy-puerperal cycle. 6 In addition, immunizing the pregnant woman is the beginning of the process of protecting this conceptus even before birth, through transplacental transfer of IgG antibodies, thereby offering passive protection for up to 12 months until the child is adequately immunized. 7 8

Unfortunately, immunization rates in pregnancy are below the desired in the world. In a study conducted in 2016, in the city of São Paulo, only 68.4% of pregnant women received the recommended vaccines. 9 Several factors may explain the poor compliance with official recommendations. Concerns about the safety of the mother or the newborn (NB) and the ignorance of vaccine recommendations by health professionals and pregnant women seem to be the most important factors for low adherence. 8

The ideal is to update the vaccine before conception, but as about half of pregnancies in the country are unplanned, Gynecologists and Obstetricians (GO) must incorporate the vaccine practice as an item of the prenatal consultation, indispensable for promoting the health of the binomial, using enlightening and compelling arguments that reinforce the adherence to vaccines by these pregnant women. The puerperal period is another moment of health care and a great opportunity to update women's vaccination. 10 11

Through bibliographic review in the PubMed database between 2010 and 2020 using the keyword “maternal immunization”, in addition to national vaccination recommendations in the pregnancy-puerperal cycle, we highlight here the main points to strengthen the knowledge and practice of GOs that assist pregnant and postpartum women, definitively incorporating immunization as a routine practice of the pregnancy-puerperal cycle.

## Why incorporate the vaccine recommendation in gynecologist and obstetrician consultations?

Because vaccines are important prevention tools and must integrate the health planning of all individuals, men and women, from birth to old age. However, vaccinating women has a range of benefits. It contributes to their protection, avoids congenital infections, allows the transmission of antibodies to the fetus, prevents the transmission of diseases to the infant and to others under their care, whether at home, in the nursery, at school, hospitals. Therefore, specific guidelines on immunization for adult women are essential. 12

Historically, there was no culture in which GOs participated effectively in immunization guidance, and only tetanus vaccination remained in pregnant women treated in the public service. In the last decade, these professionals began to be guided to participate effectively in immunization programs. 10 Initially, the licensing of HPV (human papilloma virus) vaccines in 2007 placed women as the main target population. In 2009, the dreaded H1N1 influenza pandemic occurred; pregnant women were in the risk group for complications and death, and started to be immunized routinely. The third event was the epidemic of neonatal deaths from pertussis, experienced in Brazil from 2011. The main strategy for controlling this situation is the vaccination of pregnant women at each pregnancy for the protection of the newborn, which has become a rule for the public system since the end of 2014. 10

The need to maintain high levels of vaccination coverage in the population and the use of periodic booster doses to maintain many infectious diseases controlled is well known. Gynecologists and Obstetricians are the main physicians with access to an important part of the population, women, for a long period of their lives, and must offer this periodic guidance. 10

Although the application of vaccines is not a medical act, the prescription is, remembering that every action must be documented in medical records.

## How to sensitize pregnant and postpartum women in order to increase adherence to vaccination?

Some of the reasons for poor adherence are the lack of information on the susceptibility and the greater potential for severity of some infections in pregnant women, the fear of possible side effects of vaccines, harm to the fetus, in addition to the lack of information on the beneficial potential effect achieved with maternal immunization. Obstacles, especially for the most vulnerable, are the action of anti-vaccine groups and currently, fake news disseminated through social networks and the internet. The lack of patient guidance by the attending physician, either due to ignorance or negligence, is also noteworthy. 8

The American College of Gynecology and Obstetrics 13 suggests some measures for a better maternal acceptance of vaccination:

- Education: insufficient knowledge about the susceptibility and morbidity of vaccine-preventable diseases and risks and benefits of vaccination are modifiable barriers to improve adherence to vaccination;
- Recommendation: the verbal communication provided by a physician seems to be the biggest mo tivator for the acceptance of vaccination by pregnant women;
- Standardize: obstetricians should adopt the approach to prevent maternal and child infectious diseases through vaccination as a routine or protocol in their first prenatal consultation;
- Improve convenience: obstetricians have the opportunity to consult pregnant women frequently and are seen as reliable sources of information. One way to guarantee vaccination is to offer vaccines in the same place where prenatal consultations are held.

## What vaccines are recommended during pregnancy and when should they be taken?

Influenza, hepatitis B (for those not previously immunized) and dTpa (diphtheria, tetanus and pertussis) are the vaccines indicated during pregnancy. 14

The **influenza** vaccine is recommended for every pregnant woman, every pregnancy, in any gestational phase, preferably in the period that precedes the circulation season of the influenza virus in the region. Its protection lasts between six to 12 months after application. The vaccine is also recommended for postpartum women up to 45 days after delivery, offering no risk to breastfeeding. Pregnant women have a higher risk for complications after influenza infections, namely hospitalization, admission to intensive care units and death. In addition, there is a greater risk for premature birth, low birth weight, fetal death and also risk for complications in newborns. Immunization against influenza during pregnancy-puerperal period protects the fetus in the first six months of life, since they are at greater risk of hospitalization and death from the disease, and no influenza vaccine is licensed in this age group because of the low immunogenicity of current formulations. Two vaccines are licensed and available in Brazil: in the public service, the trivalent (a strain of influenza A-H1N1, one of A-H3N2 and a variant of influenza B), and in the private service, the quadrivalent (a strain of a second strain B), which increases the protection spectrum. 14 15 16

The **dTpa** vaccine should be given after the 20 ^th^ week of pregnancy, sufficient to induce protection against neonatal tetanus in pregnant women with a previous history of complete immunization (three doses) with vaccines containing the tetanus component, or who have received two doses of dT previously. In cases of incomplete or unknown vaccination history, two doses of dT and the dTpa must be guaranteed. Women who did not receive the dTpa during pregnancy should be vaccinated in the immediate postpartum period. 10 12 15 16

Pertussis is a serious disease, where the bacteria *Bordetella pertussis* is especially virulent when it affects young infants in the first months of life. Routine dTpa vaccination during pregnancy reduces the child's risk of contracting whooping cough by approximately 90% in the first months of life. 17 18 19 This disease is transmitted through respiratory droplets from nearby infected individuals. The immunization strategy, called cocoon or cocooning, consists of the immunization of all those who live with the young infant and therefore, represent the greatest risk of transmitting the disease in the domestic environment, which is considered the main epidemic unit of the disease.

The **hepatitis B** vaccine complete schedule includes three doses (zero-one-six months) that can be started in the first trimester. If there is no previous vaccination proof, or an incomplete vaccination schedule, the orientation is to start the schedule or complete the missing doses. 16 In the absence of prophylaxis, the risk of the newborn being infected by the hepatitis B virus due to intrauterine and mainly perinatal exposure to HBsAg and HBeAg positive parturient women is 70%- 90%, falling to 5%-20% in parturient women who are HBsAg positive and HBsAg negative. Vertical transmission is associated with a higher risk for chronic infection in children. As a result, vaccination of pregnant women protects the mother from acquiring the virus during pregnancy, as well as the conceptus. 15 16 Special attention should be given to women at a higher risk for hepatitis B virus infection during pregnancy, such as: household contact members or sexual partners who are positive for hepatitis B surface antigen; more than one partner in the six months before pregnancy; recent treatment for sexually transmitted infection; current or recent injecting drug users; people living with chronic liver disease; people living with HIV; travelers to areas of high endemicity. 17

## What vaccines are given eventually during pregnancy?

Inactivated vaccines (hepatitis A, pneumococcal, meningococcal conjugate or MenACWY and meningococcal B) do not have theoretical risks for neither the pregnant woman nor the fetus. Currently, these vaccines are offered only at private clinics. Despite insufficient data on the safety of the HAV hepatitis A vaccine during pregnancy, in Brazil, there are several situations in which the risk of exposure to the virus is high. In such cases, vaccination during pregnancy should be considered, for example, women who live in inadequate sanitation conditions, or in the presence of disease outbreaks. 10 12 15

The sequential schedule with 13-valent pneumococcal and 23-valent polysaccharide conjugate vaccines should be considered in women with clinical risk factors for invasive pneumococcal disease, such as pregnant women with chronic heart disease, chronic lung disease, diabetes, chronic liver disease, cochlear implant, congenital and/or acquired immunodeficiencies, sickle cell disease or other hemoglobinopathies and anatomical or functional asplenia. 15 20 21 Similarly, mono (C) or quadrivalent (A, C, W, Y) and meningococcal B vaccines do not have safety data regarding their use during pregnancy, although in situations of epidemiological risk, the possibility of vaccination should be evaluated. 15 20 21

## Which vaccines are contraindicated during pregnancy?

The HPV vaccine and attenuated vaccines (chickenpox, triple viral - MMR and dengue), composed of live attenuated viruses, are contraindicated because they may represent a theoretical risk of transmission of the vaccine virus to the fetus. They should be recommended in the preconception period, puerperal period, in the presence or not of breastfeeding. 14 15 16

As the yellow fever vaccine consists of live attenuated virus, it is usually contraindicated in pregnant women. In situations where the risk of infection outweighs the potential risks of vaccination, it may be recommended during pregnancy. For pregnant women traveling to countries that require the International Certificate of Vaccination or Prophylaxis, they can be exempted from vaccination by the attending physician if there is no risk of contracting the infection. It is contraindicated in nursing mothers until the baby is six months old, but if vaccination cannot be avoided, breastfeeding should be suspended for ten days. 10 12 14

The dengue vaccine is contraindicated both during pregnancy and in the puerperal period. 10 12 14 15

## Which vaccines are currently being developed for use in pregnancy?

New vaccines are at different stages of development with the main purpose of preventing neonatal infectious diseases. Among them, vaccines against respiratory syncytial virus (RSV), group B streptococcus (GBS), herpes simplex virus (HSV) and cytomegalovirus (CMV) stand out as diseases for which vaccines are currently unavailable. 8 The respiratory syncytial virus is the main cause of lower respiratory tract infections in infants and children under two years of age, age groups in which infections are more severe, especially in premature newborns and infants. Premature newborns or those with underlying severe chronic heart or lung disease are at higher risk for severe RSV infection leading to hospitalization and death. As most cases of severe RSV infection occur in the first three months of life, it is unlikely that immunization of infants can provide sufficient and timely protection. Therefore, maternal immunization is considered an adequate strategy for the prevention of RSV disease in young children.

Group B streptococcus infection is a major cause of pneumonia, meningitis and sepsis in newborns. Due to the early onset of the disease, the administration of a GBS vaccine for newborns at birth does not generate an immune response quickly enough to prevent a high lethality infection. Thus, maternal immunization is identified as a potential strategy to prevent neonatal disease (early-onset disease), when associated with the use of intrapartum antibiotic prophylaxis administered to GBS positive parturient women in prenatal screening, in addition to preventing the late-onset disease (>7 to 90 days of age) as well.

Due to the risks of neonatal herpes and congenital CMV, these vaccines are being evaluated with priority for seronegative women before pregnancy. 15

## Final considerations

Women who are planning to become pregnant or who are already pregnant become more receptive to immunization, especially when informed about the goal of making the gestation period as safe and healthy as possible, and about the benefits to their baby. However, there are still low rates of adherence to prenatal vaccination, especially among pregnant women with low socioeconomic status, low education, some racial and ethnic groups, and alternative behaviors. For this reason, the theme of immunizations in preconception, pregnancy and puerperal periods must be addressed in consultations with the gynecologist, obstetrician and pediatrician. These are unique moments in women's life that should be valued by all health professionals, especially by gynecologists/obstetricians, who must include immunizations as part of their clinical practice.

National Specialty Commission for Vaccines of the Brazilian Federation of Gynecology and Obstetrics Associations (FEBRASGO)

President:

Cecília Maria Roteli Martins

Vice-President:

Nilma Antas Neves

Secretary:

Susana Cristina Aidé Viviani Fialho

Members:

André Luís Ferreira Santos

Angelina Farias Maia

Fabíola Zoppas Fridman

Giuliane Jesus Lajos

Isabella de Assis Martins Ballalai

Juarez Cunha

Júlio Cesar Teixeira

Manoel Afonso Guimarães Gonçalves

Marcia Marly Winck Yamamoto de Medeiros

Renata Robial

Renato de Ávila Kfouri

Valentino Antonio Magno
